# A Flea-Borne Mystery: Unraveling Murine Typhus in a Patient with Unexplained Encephalopathy

**DOI:** 10.1177/23247096251345086

**Published:** 2025-05-31

**Authors:** Jose Loayza Pintado, Jorge Aboytes, Cesar Uribe

**Affiliations:** 1 University of Texas Rio Grande Valley, School of Medicine, Edinburg, Texas, USA

**Keywords:** typhus, endemic flea-borne, encephalopathy, *Rickettsia typhi*, doxycycline, zoonoses

## Abstract

Murine typhus is a flea-borne rickettsial infection caused by *Rickettsia typhi*, commonly seen in endemic regions like Southern California and Texas. While it typically presents with fever, rash, and headache, neurological symptoms such as altered mental status are rare. We present a case of a 66-year-old male in southern Texas with alcohol use disorder who developed progressive confusion, decreased appetite, and subjective fevers. He was found to have severe hyponatremia, acute kidney injury, and atrial fibrillation with rapid ventricular response. Despite supportive care and empiric antibiotics for a urinary tract infection, his encephalopathy persisted. Further history revealed exposure to flea-infested cats, prompting rickettsial testing and empiric doxycycline. Typhus immunoglobulin M antibodies later confirmed the diagnosis, and the patient showed marked improvement with doxycycline therapy. Murine typhus with altered mental status is a rare presentation, often leading to diagnostic delays. This case occurred in an endemic region, with exposure to flea-infested cats as a significant risk factor. The patient’s persistent encephalopathy prompted a broad workup, including rickettsial testing, which was confirmed on serology testing. Early doxycycline initiation led to symptom resolution. This case highlights a rare neurological presentation of murine typhus and emphasizes the importance of considering it in patients with unexplained encephalopathy in endemic areas.

## Introduction

Murine typhus, also referred to as endemic or flea-borne typhus, is an acute febrile illness with nonspecific clinical features.^1^ It is caused by *Rickettsia typhi*, a Gram-negative, obligate intracellular coccobacillus. The primary reservoir for this pathogen is rat fleas and their feces, though other animals, such as cats, opossums, skunks, and raccoons, can also serve as sources of infection.^2^ Transmission occurs through various vectors, including human body lice, fleas, ticks, and ectoparasites found on flying squirrels.^3^ In certain regions of the United States, particularly Southern California and Texas, murine typhus has been resurging as a notable cause of fever. The increasing prevalence in these areas is thought to be driven by an alternative transmission cycle involving opossums and cat fleas.^4^

The clinical presentation of murine typhus commonly includes fever, reported in nearly all affected individuals (98%-100%), while about half develop a rash. A smaller subset (12.5%) presents with the classic triad of fever, headache, and rash.^5^ Although murine typhus is often considered a mild illness compared to other rickettsial diseases such as Rocky Mountain spotted fever and epidemic typhus, severe complications and fatalities can occur.^5-7^ Neurologic involvement is most frequently reported as headache, which affects approximately 81% of patients. Less common but notable neurological symptoms include stupor, ataxia, and seizures,^5^ while around 4% of cases involve altered mental status or confusion.^7^

A high level of clinical suspicion is essential for early detection of murine typhus. Indirect immunofluorescence assay remains the primary diagnostic tool. In endemic regions like Texas, a confirmed diagnosis is based on either a 4-fold increase in antibody titers between acute and convalescent samples or a single immunoglobulin M (IgM) or immunoglobulin G (IgG) titer of 1024.^8^ Polymerase chain reaction testing can also aid in confirming the diagnosis.^9,10^

Due to the delayed availability of confirmatory test results, initiating empirical treatment as soon as murine typhus is suspected is critical. Early administration of appropriate therapy not only alleviates symptoms like fever and headache but also prevents the progression to severe complications, particularly those affecting the central nervous system.^11^ Studies indicate that prompt recognition and treatment lead to shorter hospital stays.^12^ Doxycycline remains the first-line treatment for murine typhus and is recommended for all age groups.^13^

The nonspecific nature of murine typhus, particularly when neurological symptoms predominate, can complicate the diagnostic process. Here, we present a case of a patient who developed altered mental status, initially attributed to more common causes, but was ultimately diagnosed with murine typhus.

## Case Presentation

A 66-year-old male with a history of alcohol-use disorder presented to the emergency department of a southern Texas hospital with progressive confusion and decreased appetite over the past 2 days, accompanied by subjective fevers. His wife also reported noticing 1 to 2 episodes of black stools per day over the same period. On examination, the patient appeared lethargic, disoriented, and febrile, with atrial fibrillation and a rapid ventricular response.

Laboratory evaluation revealed hemoglobin of 16.3 g/dL, severe hypovolemic hypotonic hyponatremia (serum sodium 119 mEq/L, serum osmolality 268 mOsm/kg, urine osmolality 490 mOsm/kg, urine sodium 5 mEq/L), and acute kidney injury with a creatinine level of 1.45 mg/dL. Liver function tests showed elevated aspartate aminotransferase at 210 U/L, and creatine kinase was significantly elevated at 696 U/L. Cardiac markers indicated a Type II myocardial infarction with troponin levels peaking at 80 ng/L, though electrocardiography showed no ST-segment changes. Arterial blood gas analysis revealed respiratory alkalosis (pH 7.49, bicarbonate 24 mEq/L, pCO₂ 28.6 mmHg). Notably, ammonia levels were within the normal range at 30 µmol/L. Additional findings included an urinalysis remarkable for abnormal leukocyte esterase levels on 250/UL (reference: negative), few high-power field (hpf) bacteria, white blood cells 6 to 9/hpf (reference: 0-5/hpf); along with elevated inflammatory markers (erythrocyte sedimentation rate 26 mm/hour and C-reactive protein 10.7 mg/L). Toxicology screening, including ethyl alcohol levels, was negative.

**Figure 1.**
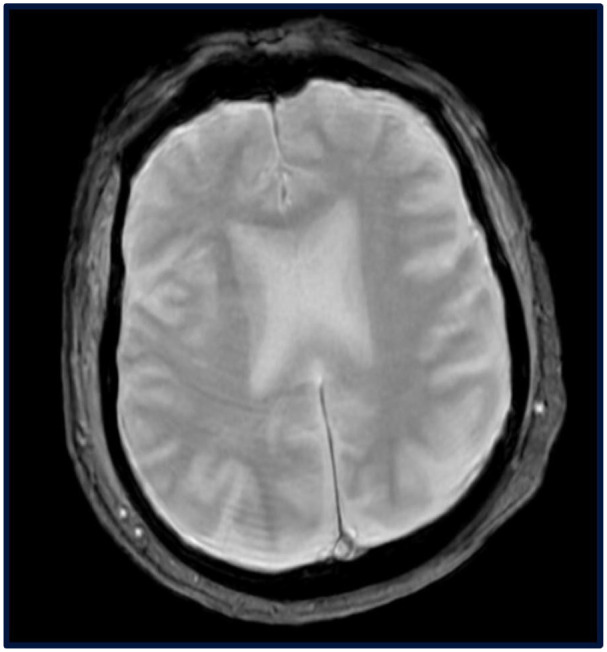
Brain MRI showing no acute infarct, abnormal signal changes, ventricular abnormalities, or extra-axial fluid collection. MRI, magnetic resonance imaging.

Imaging studies included a chest X-ray showing no acute cardiopulmonary pathology, an abdominal ultrasound revealing a heterogeneous liver with a slightly undulating contour, and liver elastography confirming cirrhosis without significant fibrosis. An echocardiogram demonstrated an ejection fraction of 50% to 54% with anterior wall hypokinesis. Neurologic imaging with computed tomography (CT) of the head and magnetic resonance imaging (MRI) of the brain showed no acute abnormalities (Figure 1).

Following admission, the patient was transferred to the intensive care unit for atrial fibrillation rate control with a diltiazem infusion after unsuccessful intravenous beta-blocker administration. Supportive care included cautious sodium correction, intravenous hydration, proton pump inhibitor therapy, and broad-spectrum empiric antibiotics with intravenous Piperacillin/Tazobactam and Vancomycin for undifferentiated severe sepsis. Gastroenterology was consulted regarding the reported melena, but given the stable hemoglobin levels, an endoscopic evaluation was deferred to the outpatient setting after stabilization.

The patient’s encephalopathy was presumed to be multifactorial, and management strategies included ruling out an acute ischemic event, lactulose for potential hepatic encephalopathy, high-dose intravenous thiamine and folic acid for suspected Wernicke’s encephalopathy, and the Clinical Institute Withdrawal Assessment (CIWA) protocol for alcohol withdrawal, utilizing chlordiazepoxide and benzodiazepines as needed. Despite these interventions, the patient’s mental status showed no significant improvement.

Further history-taking revealed that the patient had 3 cats, regularly slept with them, and had recently observed fleas on one of them. Given this exposure, testing for flea- and tick-borne infections was initiated, including rickettsial serology, and empiric doxycycline therapy was started. Neurology and infectious disease specialists were consulted due to concerns for meningoencephalitis and persistent febrile episodes. Although the patient did not exhibit signs of meningeal irritation, neurology recommended a lumbar puncture, which was declined by the patient’s wife. Given the persistent fever spikes, broad-spectrum meningoencephalitis coverage with high-dose intravenous ceftriaxone and ampicillin was initiated 2 days after doxycycline treatment began.

At this point, the patient’s fever had subsided, and his mental status showed gradual improvement. While awaiting serology results, further infectious workup, including testing for *Brucella*, West Nile virus, Lyme disease, human immunodeficiency virus, *Treponema pallidum*, and an acute hepatitis panel, returned negative. The rickettsial panel later confirmed the presence of typhus IgM antibodies (Table 1).

**Table 1.** Serology.

Serology	Result	Reference
Typhus group IgM Ab	Detected	Detected (≥1:256 Titer)/not detected
Typhus group IgG Ab	Not detected	Detected/not detected
RMSF IgM Ab	<1:64 Titer	<1:64 Titer
RMSF IgG Ab	1:128 Titer	<1:64 Titer
Lyme disease IgG Ab	Negative	Positive/negative
*Brucella* IgM and IgG Ab	Negative	Positive/negative
West Nile Virus IgM and IgG Ab	Not detected	Detected/not detected
*Treponema pallidum* Ab	Non-reactive	Reactive/nonreactive

Abbreviations: Ab, antibodies; IgG, immunoglobulin G; IgM, immunoglobulin M; RMSF, Rocky Mountain Spotted Fever.

Over the following days, the patient’s neurological status continued to improve. He became alert, oriented to person, place, and time, and was able to ambulate independently. Empiric antibiotic therapy for meningoencephalitis was discontinued after 5 days upon confirmation of rickettsial infection. Under the guidance of the infectious disease team, the patient completed a 10-day course of doxycycline and was subsequently discharged home in stable condition.

At discharge, the patient and his wife were counseled on flea control and zoonotic disease prevention, including the importance of veterinary evaluation and treatment for their household cats. They were also advised on environmental decontamination measures such as frequent vacuuming of carpets and furniture, washing bedding and pet items in hot water, and using veterinarian-approved flea control products for the home to reduce the risk of reinfection or transmission.

## Discussion

This report presents a case of murine typhus, an endemic febrile illness with global distribution, manifesting predominantly with fever and altered mental status, a presentation that has been infrequently documented in current literature. The case occurred in Southern Texas, a region where murine typhus is highly prevalent, alongside Southern California and Hawaii.^14^ Notably, the patient had a significant exposure risk, as he lived with cats, one of which was found to have fleas. The cat flea (*Ctenocephalides felis*), primarily associated with domestic cats but also found on opossums, dogs, and rats, is the leading vector in suburban areas of the United States.^15^ This underscores the importance of obtaining a thorough exposure history and considering less-common infectious etiologies rather than solely attributing encephalopathy to more typical causes, particularly in endemic areas.

Neurological manifestations of murine typhus, including confusion, encephalopathy, and altered mental status, have been reported in approximately 4% to 8% of active infections.^5,7^ A case study by Mehta et al^16^ described a pediatric patient with murine typhus presenting primarily with myositis and encephalitis, while Pervaiz et al^17^ documented a case similar to ours, involving fever and altered mental status in a young adult. Though neurological complications are uncommon, they are generally reversible, with long-term deficits rarely observed, even in untreated cases.^18^ Interestingly, severe rickettsial disease has been linked to risk factors such as alcohol use disorder, glucose-6-phosphate dehydrogenase deficiency, and the use of sulfonamide antibiotics.^19,20^ In this patient, a history of chronic alcohol use may have played a role in the severity of his neurological symptoms.

The laboratory findings were consistent with those frequently observed in murine typhus, including azotemia with proteinuria, early-stage leukopenia, thrombocytopenia, mild elevations in hepatic transaminases, hypoalbuminemia, and hyponatremia, further supporting the diagnosis.^7^ Regarding neuroimaging, cranial CT scans in murine typhus are often normal, as was the case here, though they can occasionally reveal cerebral infarctions. MRI findings in prior cases have included focal arterial infarctions, diffuse edema, meningeal enhancement, and prominent perivascular spaces.^21^ However, in this patient, MRI did not reveal any acute abnormalities.

A comprehensive evaluation of potential causes of encephalopathy was conducted. Hepatic encephalopathy was initially considered, though the patient did not improve with lactulose and had normal ammonia levels, though it is recognized that ammonia levels alone are not definitive for diagnosis.^22^ Wernicke’s encephalopathy was managed with high-dose thiamine and folic acid with which we can see some partial improvement,^23^ yet there was no significant improvement in cognitive status in the patient. Alcohol withdrawal symptoms were addressed using a regimen of scheduled chlordiazepoxide and as-needed intravenous benzodiazepines; however, altered mental status persisted beyond 5 days of alcohol cessation, a period by which withdrawal symptoms typically resolve.^24^ It is also noteworthy that the patient’s CIWA score remained below 8, indicating only mild withdrawal symptoms.

Although a lumbar puncture was recommended by Neurology, it was ultimately declined by the patient’s next of kin. Notably, prior studies have shown that even in cases where fever, nuchal rigidity, and photophobia are prominent, cerebrospinal fluid (CSF) analysis may appear normal. When indicative of meningoencephalitis, CSF findings often resemble those of viral etiologies.^25^

This case highlights the importance of considering murine typhus in the differential diagnosis of altered mental status, particularly in endemic regions where flea-borne diseases are prevalent. While fever and rash are the hallmark symptoms of murine typhus, neurological involvement, though rare, can present a significant diagnostic challenge, leading to delays in treatment. Given that early initiation of doxycycline can improve symptoms in a short period of time and prevent complications, maintaining a high index of suspicion for rickettsial infections in patients with zoonotic risk factors and unexplained encephalopathy is crucial. This case serves as a reminder that a thorough history, including environmental and animal exposures, remains a cornerstone in identifying atypical infectious etiologies, ensuring timely and effective treatment.

## References

[1] Blanton LS Dumler JS Walker DH. Rickettsia typhi (Murine Typhus). Elsevier eBooks; 2015.

[2] Civen R Ngo V. Murine typhus: an unrecognized suburban vectorborne disease. Clin Infect Dis. 2008;46(6):913-918. 18260783 10.1086/527443

[3] Biggs HM Behravesh CB Bradley KK , et al. Diagnosis and management of tickborne rickettsial diseases: Rocky Mountain spotted fever and other spotted fever group rickettsioses, ehrlichioses, and anaplasmosis—United States. MMWR Recomm Rep. 2016;65(2):1-44. 10.15585/mmwr.rr6502a1 27172113

[4] Azad AF Radulovic S Higgins JA , et al. Flea-borne rickettsioses: ecologic considerations. Emerg Infect Dis. 1997;3(3):319-327. 9284376 10.3201/eid0303.970308 PMC2627639

[5] Dumler JS. Clinical and laboratory features of murine typhus in South Texas, 1980 through 1987. JAMA. 1991;266(10):1365. 1880866

[bibr6-23247096251345086] Pieracci EG Evert N Drexler NA , et al. Fatal flea-borne typhus in Texas: a retrospective case series, 1985-2015. Am J Trop Med Hyg. 2017;96(5):1088-1093. 28500797 10.4269/ajtmh.16-0465 PMC5417200

[7] Tsioutis C Zafeiri M Avramopoulos A , et al. Clinical and laboratory characteristics, epidemiology, and outcomes of murine typhus: a systematic review. Acta Trop. 2016;166:16-24. 27983969 10.1016/j.actatropica.2016.10.018

[8] Murray KO Evert N Mayes B , et al. Typhus group Rickettsiosis, Texas, USA, 2003-2013. Emerg Infect Dis. 2017;23(4):645-648. 28322701 10.3201/eid2304.160958 PMC5367421

[9] Theunissen C Cnops L Van Esbroeck M , et al. Acute-phase diagnosis of murine and scrub typhus in Belgian travelers by polymerase chain reaction: a case report. BMC Infect Dis. 2017;17(1):273. 28407761 10.1186/s12879-017-2385-x PMC5390359

[10] Paris DH Dumler JS. State of the art of diagnosis of rickettsial diseases: the use of blood specimens for diagnosis of scrub typhus, spotted fever group rickettsiosis, and murine typhus. Curr Opin Infect Dis. 2016;29(5):433-439. 27429138 10.1097/QCO.0000000000000298 PMC5029442

[11] Newton PN Keolouangkhot V Lee SJ , et al. A prospective, open-label, randomized trial of doxycycline versus azithromycin for the treatment of uncomplicated murine typhus. Clin Infect Dis. 2018;68(5):738-747. 10.1093/cid/ciy563 PMC6376095 30020447

[12] Howard A Fergie J. Murine typhus in South Texas children. Pediatr Infect Dis J. 2018;37(11):1071-1076. 29465481 10.1097/INF.0000000000001954

[13] Heymann D.L. Control of Communicable Diseases Manual. American Public Health Association; 2015.

[14] Centers for Disease Control and Prevention. Flea-Borne (Murine) Typhus. Centers for Disease Control and Prevention; 2023.

[15] Adams WH Emmons RW Brooks JE. The changing ecology of murine (Endemic) typhus in Southern California. Am J Trop Med Hyg. 1970;19(2):311-318. 4986201 10.4269/ajtmh.1970.19.311

[16] Mehta M Marek R Arthur C , et al. Localized myositis and transient encephalopathy as presenting symptoms in murine typhus. Pediatr Infect Dis J. 2024;43(7):e242-e244. 10.1097/INF.0000000000004306 38451920

[17] Pervaiz AM Tariq R Bangash SA Lal Y. Murine typhus presenting with acute psychosis and disseminated intravascular coagulation: A case report. Cureus. 2019;11(4):e4450. 10.7759/cureus.4450 PMC6561526 31205836

[18] Carr SB Bergamo DF Emmanuel PJ , et al. Murine typhus as a cause of cognitive impairment: case report and a review of the literature. Pediatr Neurol. 2013;50(3):265-268. 24321542 10.1016/j.pediatrneurol.2013.09.017

[19] Whelton A. Acute renal failure complicating rickettsial infections in glucose-6-phosphate dehydrogenase-deficient individuals. Ann Intern Med. 1968;69(2):323. 5695625 10.7326/0003-4819-69-2-323

[20] Walker DH. The role of host factors in the severity of spotted fever and typhus rickettsioses. Ann N Y Acad Sci. 1990;590(1):10-19. 2198829 10.1111/j.1749-6632.1990.tb42201.x

[21] Xu Z Zhu X Lu Q , et al. Misdiagnosed murine typhus in a patient with multiple cerebral infarctions and hemorrhage: a case report. BMC Neurol. 2015;15:121. 26223226 10.1186/s12883-015-0383-4 PMC4520183

[22] Vilstrup H Amodio P Bajaj J , et al. Hepatic encephalopathy in chronic liver disease: 2014 Practice Guideline by the American Association for the Study of Liver Diseases and the European Association for the Study of the Liver. Hepatology. 2014;60(2):715-735. 25042402 10.1002/hep.27210

[23] Vasan S Kumar A. Wernicke Encephalopathy. StatPearls Publishing; 2023. 29261914

[24] Jesse S Bråthen G Ferrara M , et al. Alcohol withdrawal syndrome: mechanisms, manifestations, and management. Acta Neurol Scand. 2016;135(1):4-16. 27586815 10.1111/ane.12671 PMC6084325

[25] Dittrich S Rattanavong S Lee SJ , et al. Orientia, rickettsia, and leptospira pathogens as causes of CNS infections in Laos: a prospective study. Lancet Glob Health. 2015;3(2):e104-e112. 10.1016/S2214-109X(14)70289-X PMC4547322 25617190

